# Salovum^®^ reduces mortality in isolated severe traumatic brain injury: a randomized phase II trial

**DOI:** 10.1093/brain/awag241

**Published:** 2026-09-07

**Authors:** David Cederberg, Bradley M Harrington, Iain Walker, Ruan Grobler, Nitesh Naranbhai, Ryan K Jacobs, Edward Visse, Niklas Marklund, Adriaan Johannes Vlok, Peter Siesjö

**Affiliations:** Department of Clinical Sciences Lund, Neurosurgery, Lund University and Skane University Hospital, Lund 222 42, Sweden; Department of Neurosurgery, Tygerberg University Hospital, Cape Town 7505, Republic of South Africa; Department of Neurosurgery, Tygerberg University Hospital, Cape Town 7505, Republic of South Africa; Department of Neurosurgery, Tygerberg University Hospital, Cape Town 7505, Republic of South Africa; Department of Neurosurgery, Tygerberg University Hospital, Cape Town 7505, Republic of South Africa; Department of Neurosurgery, Tygerberg University Hospital, Cape Town 7505, Republic of South Africa; Department of Clinical Sciences Lund, Neurosurgery, Lund University and Skane University Hospital, Lund 222 42, Sweden; Department of Clinical Sciences Lund, Neurosurgery, Lund University and Skane University Hospital, Lund 222 42, Sweden; Department of Neurosurgery, Tygerberg University Hospital, Cape Town 7505, Republic of South Africa; Department of Clinical Sciences Lund, Neurosurgery, Lund University and Skane University Hospital, Lund 222 42, Sweden

**Keywords:** TBI, randomized controlled trial, neuroprotection, neurointensive care, secondary brain injury, neurosurgery

## Abstract

Isolated severe traumatic brain injury (TBI) is associated with high mortality and long-term morbidity, especially in low- and middle-income countries (LMICs). Despite promising results using pharmacological and non-pharmacological therapies in the experimental setting, no clinical trials have demonstrated robust efficacy. We aimed to investigate the effect of antisecretory factor (Salovum^®^) on 30-day mortality in adult patients with severe TBI.

This was a prospective, double-blind, placebo-controlled phase 2 trial, conducted from September 2017 to February 2022 in a tertiary trauma centre (university hospital) in Cape Town, South Africa (www.clinicaltrials.gov: NCT03339505). The participants were adults with isolated severe TBI (Glasgow Coma Score 6–8), receiving intracranial pressure (ICP) monitoring and neurointensive care.

Participants were administered either Salovum^®^ (freeze-dried egg yolk powder enriched with the active compound antisecretory factor) or placebo (freeze dried egg yolk powder) via the nasogastric tubing, dosed according to weight (10–17 g/4 h) and initiated at 32.9 h ± 10.7 h (mean ± standard deviation) post-injury. Study drug or placebo were administered until the ICP monitor was removed or for a maximum of 5 days.

The primary outcome of this trial was 30-day mortality and secondary outcome measures were therapy intensity level (TIL) and ICP.

One hundred participants aged 18–61 were included, with 49 and 51 patients in the treatment and control groups, respectively. Most patients were male (96%) and the mean age was 33 years. The 30-day mortality rate was 20% in the treatment group and 39% in the control group, yielding a relative risk ratio of 0.52 (confidence interval: 0.271–0.997) and a *P*-value of 0.040 (chi-square test). TIL and ICP were similar in both groups. No significant adverse events were noted.

Antisecretory factor significantly reduced 30-day mortality in severe TBI in an LMIC environment. Further studies investigating the mechanisms by which antisecretory factor act are warranted. The safety, easy administration and marked efficacy of antisecretory factor support exploration in multicentre randomized controlled trials.

## Teaser

Cederberg *et al.* present the results of a double-blind, placebo-controlled trial in patients with severe traumatic brain injury, showing that treatment with Salovum^®^—a medical food supplement enriched with antisecretory factor—almost halved the 30-day mortality rate. The findings support further evaluation of Salovum^®^ in larger trials.

## Introduction

Traumatic brain injury (TBI) is a global occurence with a significant impact on society in terms of human suffering and health care costs. Worldwide, more than 70 million people experience a TBI each year, and over 2 million die as a result.^1,2^ While advances in neurointensive care, cerebral monitoring and neuroradiology have improved outcome for patients with severe TBI, global results remain poor, with reported mortality ranging from 27% to 70% and substantial long-term morbidity.^3-5^

The most commonly used definition of severe TBI is a reduced level of consciousness, e.g. a Glasgow Coma Score (GCS) score of ≤8 at admission.^6^ Patients presenting with an admission GCS score of eight or less have the highest mortality and morbidity rates, and a markedly reduced life expectancy.^7,8^

Outcome following severe TBI has been reported at multiple time points, including in-hospital and short-term mortality (e.g. 28–30 days), as well as longer-term outcomes at 6 and 12 months or beyond, reflecting the dynamic recovery trajectory after injury.^9^ Although there is no consensus on the most appropriate method, 28- and 30-day mortality are regarded as direct measures of immediate hospital intervention in moderate–severe TBI, while later follow up aims to evaluate the long-term consequences of TBI.^10,11^

The initial injury results in a primary injury that includes injury to vascular, neuronal and glial cells and often results in extra- and intracerebral haemorrhages. This initial primary injury is followed by secondary events such as oedema, vasospasm, hypoperfusion, spreading depolarization,^12^ mitochondrial dysfunction,^13^ inflammation and excitotoxicity, in addition to long-term progressive neurodegeneration.^14^ One common early denominator in all forms of severe TBI is cerebral oedema, which may result in raised intracranial pressure (ICP) which is considered a major cause of mortality and morbidity.^15^ While uncontrollable ICP is a key contributor to mortality in severe TBI, the incidence has diminished as treatment algorithms aiming to control ICP have been widely implemented.^16,17^

The pathophysiological mechanisms of traumatic cerebral oedema are only partially known and although several experimental reports and early clinical trials using various pharmacological compounds and non-pharmacological interventions have claimed beneficial effects, all phase 3 trials to date have failed.^18,19^

Antisecretory factor (AF) is a 41 kDa endogenous proteasomal protein proposed to possess both antisecretory and anti-inflammatory effects. AF, also known as PSMD4, Rpn10 and S5a recognizes polyubiquitylated proteins in the 19S proteasome regulatory particle and recruits them for degradation by the 20S core particle.^20^

The exact mechanism of soluble AF or AF-derived peptides is unknown, but experimental studies have implied an effect on the NKCC1 ion pump, also implicated in the evolution of the breakdown of the blood–brain barrier and oedema formation in TBI.^21-23^ Furthermore, PSMD4 has been reported to regulate inflammation by effects on the transcription factor NF-κB.^24^

Salovum^®^ is an egg yolk powder with high concentrations of AF and AF-derived peptides and classified as a food supplement for specific medical purposes (FSMP) in the EU and US. Salovum^®^ has been used in clinical trials for gastroenteritis,^25^ Meniere’s disease^26^ and inflammatory bowel disease^27^ with no toxicity reported. An active part of AF has been identified and synthesized into a 16 amino acid peptide; AF-16. AF-16 has shown effects against cerebral oedema and increased ICP in an experimental model of herpes encephalitis and experimental models of traumatic and cryogenic brain injury where treatment reduced mortality and normalized intracranial pressure.^28-31^ Two clinical case series have reported the use and effects of Salovum^®^ administration in adults with severe TBI demonstrating reduction of elevated ICP after administration of Salovum via a nasogastric tube or rectally.^32,33^ Probably because the half-life of AF-16 and other active peptides is short, no reliable method to measure AF or AF-derived peptides in blood or CSF is currently available.

Based on these initial findings, we designed a trial to evaluate the efficacy of Salovum^®^ in adults with severe TBI. The primary outcome of this trial was 30-day mortality, and the secondary outcomes were therapy intensity level (TIL) and ICP.^34^

## Materials and methods

### Trial design

The full research protocol has been published previously.^34^ The trial is a single-centre, investigator-initiated, prospective, double-blind, placebo-controlled randomized trial conducted at the Department of Neurosurgery, Tygerberg University Hospital, Cape Town, South Africa. The trial was designed by the sponsors (academically sponsored by Peter Siesjö and David Cederberg, Skane University Hospital, Lund, Sweden), conducted by the investigators and monitored by an external monitoring body. The trial was supported by unrestricted grants from Lantmännen AB, Stockholm, Sweden, and research grants from Lund University and Skane Region, Sweden. The funders had no influence on the design or conduct of the trial, and were not involved in data collection, analysis, the writing of the manuscript nor the decision to submit it for publication. The trial protocol (available as Supplementary material) was approved by the regional ethics committee, Human research ethics committee (HREC M16/10/040) Stellenbosch University, Stellenbosch, South Africa, and conducted in accordance with the Declaration of Helsinki. Because of a misunderstanding, one study participant was recruited on 18 September 2017, which was 2 days before the final submission release of the research protocol on www.clinicaltrials.gov (NCT03339505), thus included before the release of the protocol. To avoid bias when recruiting from multiple centres with different treatment algorithms and referral patterns^4,35^ we chose to conduct the trial in a single tertiary TBI centre.

### Participants

Adults ≥18 years old with severe TBI and a GCS of 6–8 after resuscitation, admitted to the neurointensive care unit at Tygerberg Academic Hospital and receiving invasive ICP monitoring, were recruited between September 2017 and February 2022. Although the trial was registered with inclusion criteria of GCS >3 and <9, the protocol was revised prior to recruitment to include only patients with GCS 6–8, reflecting local admission practices at the participating site. Initially, permission from next of kin was required before inclusion of participants (Supplementary material). In December 2018, an amendment to the ethical application was approved, stating that recruitment of participants for the trial could be performed with delayed consent from next of kin (Supplementary material). A complete list of inclusion and exclusion criteria is provided in the Supplementary material.

### Adverse events and serious adverse events

Adverse events relevant in clinical trials of severe TBI such as ventilator-acquired pneumonia, seizures, other infections, diarrhoea, deep venous thrombosis, pulmonary embolism and abnormal laboratory test results were recorded. Adverse events were noted according to the nomenclature of the Common Terminology Criteria for Adverse Events (CTCAE) version 5.0 with the respective grades of seriousness noted.

### Standard treatment

The Neurointensive Care Unit (NICU) at Tygerberg Hospital follows a standardized treatment protocol aligned with the Brain Trauma Foundation guidelines. The primary goal is to manage ICP using the following interventions:

- 30° head elevation to promote venous drainage.
- Tiered sedation, starting with propofol for sedation and morphine for analgesia, with midazolam added for deeper sedation if required. Barbiturates are reserved as third-tier therapy.
- Hyperosmolar therapy using either mannitol or hypertonic saline bolus.
- CSF drainage, which is frequently employed for ICP control.
- Surgical interventions, including evacuation of mass lesions and decompressive hemicraniectomy, performed either alongside primary surgery or when medical treatments fail to control ICP.

### Trial procedures

Participants were randomly assigned in a 1:1 ratio to either the Salovum^®^ group or the control group. Block randomization with block sizes ranging from four to six was used.

The participants were assigned to receive either Salovum^®^ or placebo egg yolk powder. The trial substance was packaged into boxes marked with the randomization number only. Each box contained enough substance to supply a participant with the highest dosage of substance for 5 days. Salovum^®^ and placebo egg yolk powder were indistinguishable, ensuring that both participants and investigators were blinded. The trial substance was weighed and mixed with water before administration to the participants by the nursing staff.

Participants were administered the substance for a maximum of 5 days, or until the ICP monitor was removed, if this occurred earlier. Based on previous clinical trials and case series in TBI, the substance was administered as 10 g/dosage (participants <60 kg), 14 g/dosage (participants 60–80 kg) and 17 g/dosage (participants >80 kg) via a nasogastric feeding tube every 4 h. During the trial, ICP was recorded hourly by a Camino fiberoptic intraparenchymal ICP monitor. Mean arterial blood pressure (MAP) was recorded hourly. The TIL^36^ was summarized and recorded for every 24 h period (Supplementary material).

### Outcomes

The primary outcome was 30-day mortality. The secondary outcomes were mean ICP in mmHg over the treatment period and TIL per day over the treatment period. All safety outcomes were based on adverse event reporting. The trial was monitored by an external monitoring body (Novotech Pty Limited ACN 071 874 881, 235 Pyrmont Street, Pyrmont, NSW 2009, Australia). An independent safety and monitoring committee (DMC) performed an unblinded interim analysis on 93 included participants. The DMC recommended continuing the trial with additional participants, but the sponsor decided to finalize the trial at the prescribed 100 participants due to lack of funding. Plasma samples were collected from participants if consent was given by the next of kin. The samples were intended for exploratory end points and are thus not addressed in the present trial.

### Statistical analysis

Baseline comparisons between the groups were computed using the Mann–Whitney U-test or Wilcoxon test for continuous data and chi-square test for binary data. Characteristics for TBI-IMPACT (International Mission for Prognosis and Analysis of Clinical Trials in TBI) calculations were retrieved from the patient charts.^37^ Complete data were available for 81 patients (41 in the placebo group and 40 in the intervention group). To address missing data, multiple imputation (*n* = 5) was performed. Risk estimates from the complete data set (*n* = 81) and the imputations were compared using the Wilcoxon test.

Based on previous case series, a formal sample size estimate of 100 participants was made using the chi-squared power test (pwr.chisq.test in R). An estimated sample size of 100 participants would allow us to detect a reduction in mortality of 25 percentage points, a reduction in ICP of 5 mmHg and a reduction of 5 points on the TIL scale. We extended a *post hoc* analysis to time over ICP 22, 25 and 30 mmHg. Comparisons within groups before and after study intervention were also prespecified but omitted from the results, since very few data points were available prior to study intervention. The 30-day mortality was calculated with the prespecified chi-square test, but calculation of relative risk was also added as this method is recommended for binary outcomes, i.e. mortality. Relative risk ratios with confidence intervals were visualized with a forest plot. ICP and TIL comparisons were calculated using the non-parametric Mann–Whitney U-test, where *P* < 0.05 was considered statistically significant. All computations were performed with the free R software.^38^

## Results

### Participant demographics

From 22 September 2017, a total of 100 participants with isolated TBI were enrolled (Fig. 1); 49 participants were randomly assigned to the Salovum^®^ group and 51 participants to the control group.

**Figure 1:**
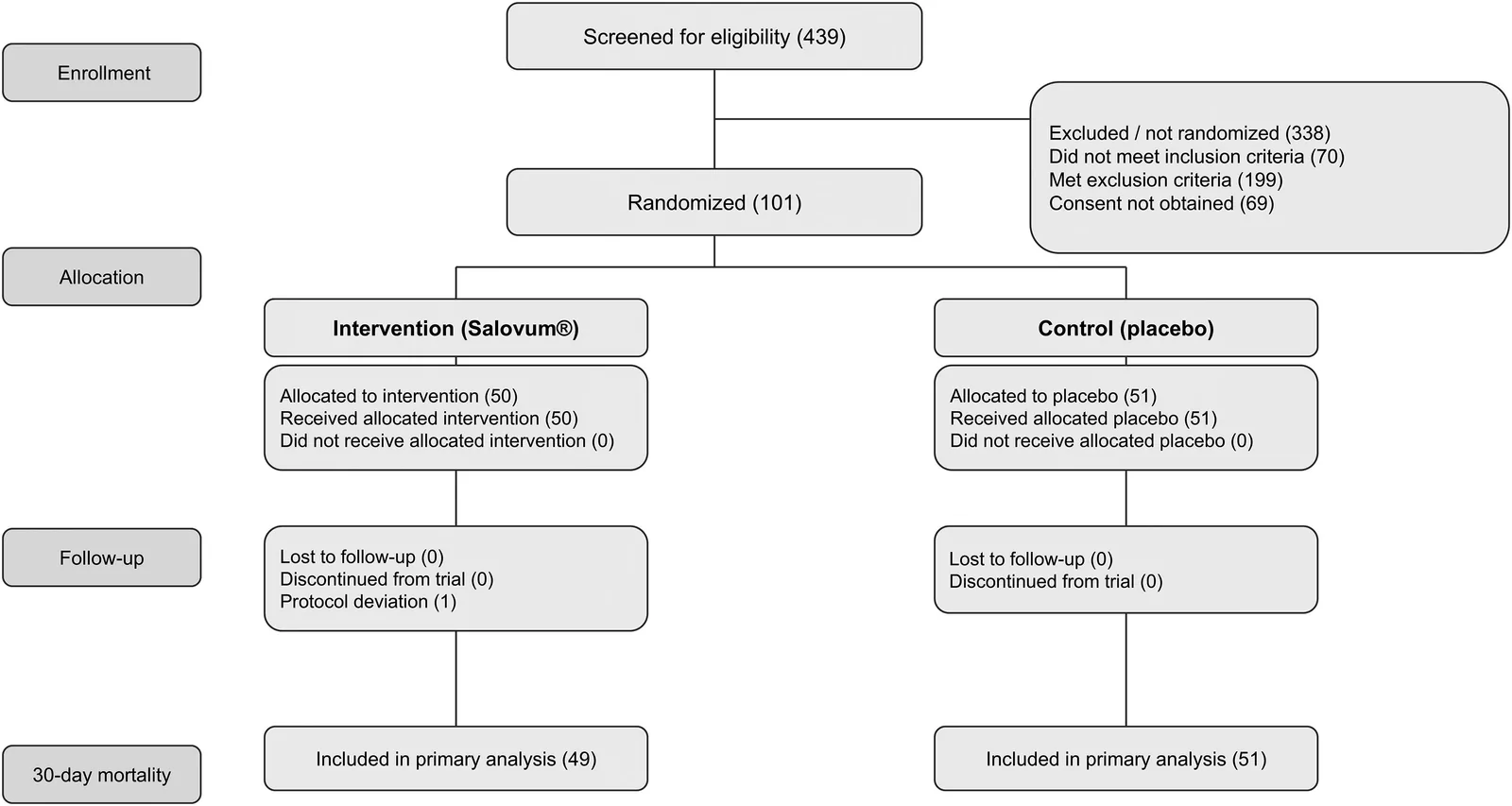
**CONSORT diagram.** Main reasons for not meeting inclusion criteria: age <18 (*n* = 63), age unknown (*n* = 7). Main reasons for meeting exclusion criteria after initial inclusion procedure: Glasgow Coma Scale >8 (*n* = 81), age >65 (*n* = 1), >24 h from trauma (*n* = 27), epidural haematoma only (*n* = 18), polytrauma (*n* = 8), transferred to private care (*n* = 1), penetrating brain injury (*n* = 63). No consent: (*n* = 69).

Male gender was predominant (96%), and the mean age was 33 years. TBI subtypes were distributed into no mass lesion (2%), subdural haematomas (23%), isolated contusions (41%) and mixed trauma types (34%). There were no significant differences in baseline characteristics, including age, gender and trauma type, between the groups (Table 1). There were no significant differences between TBI-IMPACT characteristics or risk estimates (Table 2 and Supplementary Table 1).

**Table 1:** Baseline characteristics

	Active	Placebo	*P*-value (chi-square)
Patients	49	51	NA
Age	32.6	32.8	0.52
Sex, female/male	1/48 (2%/98%)	3/48 (6%/94%)	0.638
**Type of injury**			
Contusion	17/49 (35%)	24/51 (47%)	0.728
Subdural haematoma	12/49 (24%)	11/51 (24%)	0.327
Traumatic subarachnoidal haemorrhage	8/49 (16%)	3/51 (6%)	0.095
No mass lesion	1/49 (2%)	1/51 (2%)	0.396
Mixed	19/49 (39%)	15/51 (29%)	0.177

NA = not applicable.

**Table 2:** Comparison TBI-IMPACT characteristics and risk estimates

	Active (*n* = 49)	Placebo (*n* = 51)	Missing values (active/placebo)	*P*-value^a,b^
**TBI IMPACT characteristics**				
Age	32.6	32.8	0	0.52
GCS motor score	–	–	1/2	0.43
2	0	1	–	–
3	6	2	–	–
4	13	14	–	–
5	28	29	–	–
6	1	3	–	–
Pupils	–	–	7/11	0.88
Normal	29	27	–	–
One dilated	13	13	–	–
Both dilated	0	0	–	–
Hypoxia	3	0	1/3	0.48
Hypotension	1	1	1/3	1
CT class TBI-IMPACT	–	–	0/1	0.62
II	10	12	–	–
III–IV	15	17	–	–
V–VI	24	21	–	–
tSAH	8/49 (16)	3/51 (6)	–	0.095
EDH	8/49	6/51	–	0.25
Glucose	7.4	7.9	–	0.73
Hb	12.7	12.5	–	0.46
TBI-IMPACT risk estimates				
6-month mortality risk	13.5	13.4	8/11	0.73
6-month unfavourable outcome risk	26.5	26.5	8/11	0.73

EDH = epidural haematoma; GCS = Glasgow Coma Scale; Hb = haemoglobin; TBI-IMPACT = International Mission for Prognosis and Analysis of Clinical Trials in Traumatic Brain Injury; tSAH = traumatic subarachnoid haemorrhage.

^a^Wilcoxon test.

^b^Chi-square test.

### Outcome

#### 30-Day mortality

The 30-day mortality was 10/49 participants in the Salovum^®^ group and 20/51 participants in the control group. This difference was statistically significant (*P* = 0.040) using the chi-squared test, and the relative risk ratio was 0.52 [confidence interval (CI): 0.271–0.997]. Relative risk estimates for subgroups defined by trauma mechanism suggested a lower risk for subdural haematoma compared with contusions; however, the CIs crossed 1, indicating that these differences were not statistically significant (Fig. 2).

**Figure 2:**
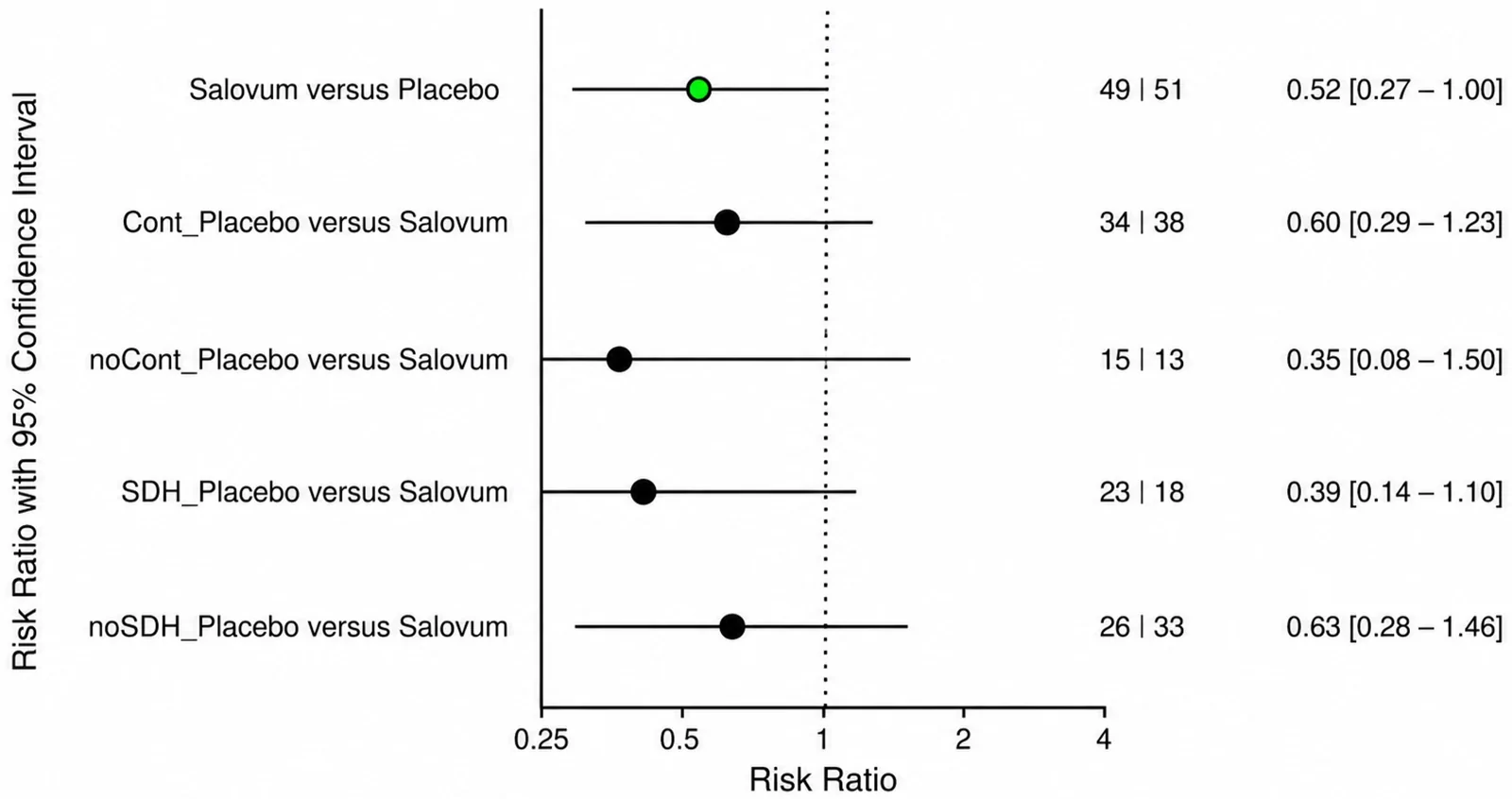
**Forest plot of relative risk ratio for 30-day mortality with confidence intervals for the whole population and subgroups according to trauma mechanisms**. Cont = contusions; SDH = subdural haematoma.

#### Intracranial pressure and therapy intensity level

ICP in the Salovum^®^ group and control group was measured as mean ICP and/or total time with ICP above thresholds of 20, 22, 25 and 30 mmHg. Mean ICP for the duration of the intervention period was <20 mmHg in both groups. There were no significant differences between the treatment groups for any of the ICP thresholds (Table 3).

**Table 3:** Trial characteristics

	Active	Placebo	Total	*P*-value
Patients, *n*	49	51	100	NA
NICU stay (days), mean ± SD	10.2 ± 4.5	10.5 ± 6.7	10.4 ± 5.7	0.735
Hospital stay (days), mean ± SD	28.1 ± 20.4	28.3 ± 32.3	28.2 ± 26.9	0.382
Trial dose, g	14.6 ± 1.2	14.8 ± 1.4	14.7 ± 1.2	0.472
Time to intervention, h; mean ± SD	32.9 ± 11.6	32.9 ± 8.7	32.9 ± 10.1	0.509
TIL d1, median (IQR)	6 (4–9)	7 (4–8)	6.0 (4–9)	0.753
TIL d2, median (IQR)	6 (4–9)	6 (3–8)	6 (3–9)	0.731
TIL d3, median (IQR)	5.8 (3–8)	8 (3.5–9)	6 (3–9)	0.103
TIL d4, median (IQR)	5 (2.8–9)	7 (5–8)	6 (3–8.5)	0.206
TIL d5, median (IQR)	5.5 (2.8–8.3)	7.5 (6–8)	6.5 (4.8–8)	0.184
TIL total	6 (3.5–8.8)	6.5 (3–8)	6 (3.3–8)	0.872
DC, *n* (%)^a^	16/49 (33)	20/5 (39)	36/100	NS
ICP > 20, h, mean ± SD	30.8 ± 29.2	30.8 ± 29.7	30.8 ± 29.3	0.0978
ICP > 22, h, mean ± SD	21.9 ± 25.8	24.4 ± 26.7	23.0 ± 26.2	0.565
ICP > 25, h, mean ± SD	12.8 ± 18.1	14.5 ± 19.1	13.7 ± 18.5	0.526
ICP > 30, h, mean ± SD	4.8 ± 8.6	5.6 ± 12.9	5.2 ± 11.0	0.0759
ICP total	19.4 ± 6.5	18.7 ± 6.3	19.0 ± 6.4	0.484
CPP, mean ± SD	72.0 ± 9.7	72.0 ± 10	72.1 ± 9.8	0.866
Treatment days, mean ± SD	3.8 ± 1.1	3.5 ± 1.3	3.6 ± 1.2	0.144
ICP monitoring ended^a^	29/4/16	31/2/18	60/6/34	0.811
Mortality	20%	39%	30/100	–
Relative risk	–	–		0.52 (0.271–0.997)
Chi-square	–	–		*P* = 0.040

CPP = cerebral perfusion pressure; DC = decompressive craniectomy; ICP = intracranial pressure; IQR = interquartile range; NA = not applicable; NICU = neurointensive care unit; NS = not significant; SD = standard deviation; TIL = treatment intensity level.

^a^Controlled ICP/uncontrolled ICP/unknown reason.

In the original SAP, analysis of difference between alive and dead participants, and within trial groups, in ICP and TIL were specified but showed no significant differences.

There were no significant differences between the Salovum^®^ and control group for total TIL during the trial period or for daily TIL during treatment (Table 3). However, in those surviving to 30-days post-injury, the median TIL on Day 3 was 4 in the Salovum^®^ and 8 in the control group (*P* = 0.041).

#### Decompressive craniectomies and treatment days

The vast majority of decompressive craniectomies (DC) (34/36) were primary, and there was no significant difference between the number of DC performed in the groups (Salovum^®^ 16/49, control 20/51; Table 3).

There was no significant difference in the number of treatment days. The number of treatment days was 3.8 in the Salovum^®^ group and 3.5 in the control group, since treatment was discontinued if the ICP monitor was removed prior to the end of the 5-day treatment duration.

#### Safety

Adverse events and serious adverse events were noted in the case report form daily by the physician responsible for the participant. The daily chart states: ‘Do you consider that there is a reasonable possibility that an adverse event has been caused by the study compound?’ This question was answered daily.

Adverse events caused by the trial substance were defined as skin rash and hives. Serious adverse events were defined as a serious anaphylactic reaction with hypotension and bronchospasm, requiring medical intervention with corticosteroids and/or vasopressors. No adverse event of these sorts were reported. Anaphylactic allergic reactions to egg yolk are, according to the literature, extremely rare.^39^

To investigate the difference in adverse events and serious adverse events between the groups, the investigators reviewed patients’ charts and investigated all study participants retrospectively.

The dominating adverse event was pneumonia. There was no significant difference in the incidence of adverse events between the groups. (Table 4)

**Table 4:** Adverse event reporting

Adverse event^a^	Grade^b^	Placebo	Active	*P*-value^c^
Pneumonia	3	24	26	0.55
Seizure	4	2	2	0.24
Creatinine increased	1	3	2	0.68
Liver enzymes increased	2	1	1	0.98
Diarrhoea	1	2	2	0.97
Cardiac troponin T increased	1	1	2	0.53
Urinary tract infection	3	2	0	ND
Skin infection	3	1	0	ND

ND = not determined.

^a^According to Common Terminology Criteria for Adverse Events (CTCAE) 5.0.

^b^According to CTCAE 5.0.

^c^Chi-square statistics.

One participant was unblinded and excluded from the trial because the trial substance could (accidentally) not be located for the study. This participant, who was administered active compound, was not included in the per protocol analysis but was included in the intent to treat analysis for the primary aim. The intent to treat analysis, thus including 50 participants with active compound and 51 with placebo, showed a relative risk for mortality of 0.51 (CI: 0.266–0.978) for the intervention.

## Discussion

In this randomized, controlled and double-blinded single-centre trial, oral administration of Salovum^®^, an egg yolk powder containing increased amounts of the endogenous protein AF, significantly reduced mortality in participants with severe TBI.

Multiple randomized controlled trials on patients with severe TBI have failed to significantly reduce mortality, morbidity or improve long-term outcome using interventions such as hypertonic saline, erythropoietin, progesterone, tranexamic acid and hypothermia.^40^ For decompressive craniectomy, one trial succeeded in showing a significant decrease in mortality at 6 months.^41^ Most recently, the CRASH III trial, which assessed and included participants with moderate and severe TBI who were randomized into either receiving tranexamic acid or a placebo, did not show a significant difference in total. However, subgroup analysis showed an effect on 30-day mortality in participants with moderate TBI,^10^ tentatively attributed to a reduction of secondary haemorrhagic events in this participant group. The administration of tranexamic acid had no effect on mortality in participants with severe TBI.

Randomized controlled trials (RCTs) in TBI are hampered by several factors such as heterogeneity with respect to both types of trauma, recruitment policy, catchment area and transfer times from primary hospitals to admission. In tertiary NICUs, off-label use of pharmacological and non-pharmacological interventions is frequent, outside the prevailing treatment algorithms.^42^ In addition to varying treatment protocols in different centres, alternative thresholds for when to initiate tier treatment exist. Performing the trial at a single site potentially reduces inter-site confounders that may have a greater impact on the outcome than the intervention being tested. Thus, we conducted the trial in a single, tertiary TBI centre with a high caseload of severe TBI.

AF is an endogenous proteasomal protein found in humans and many other species. When administered to humans and rodents as whole protein and peptides, it has several potential mechanisms of action. These effects could originate from either proteasomal modulation or mechanisms outside the proteasome. However, unlike other proteasome modulators, no side effects of Salovum^®^ have been noted in various reports, including two case series of severe TBI. In the present trial, 49 participants were administered Salovum^®^ for a median of 3.8 days without any signs of adverse events, which is in line with previous reports.^32,33^ In a phase 1–2 trial of participants with primary glioblastoma, Salovum^®^ was given at an equivalent dose (1 g/kg) for more than 50 days without signs of side effects or toxicity, further strengthening the safety profile of the intervention.^43^

To assess potential confounding, baseline and treatment characteristics were compared between groups, and no significant differences were identified. In addition, predicted risks of 6-month mortality and unfavourable outcome, based on the TBI-IMPACT model, were evaluated and found to be similar between groups. These findings suggest that the groups were well balanced and support the interpretation that the observed differences in outcome are attributable to the intervention.

Hospital and post-treatment mortality rates (hospital case fatality) of severe TBI patients vary substantially between 10% and 70% in reports from retrospective studies, RCTs and the very few population-based studies.^44^ This notion makes comparisons and reference data ambiguous depending on inclusion and exclusion criteria. In RCTs, additional causes of non-representativeness can also exist, such as exclusion after prior inclusion, standard or logistics-based exclusion criteria from centres involved or that eligible participants are not admitted at the trial centre due to logistic reasons. In the present trial, mortality at 30 days was 20% in the Salovum^®^ group and 39% in the control group. The mortality in the control group corresponds well with the numbers from large, randomized trials from low-income countries such as the CRASH trial and available data from South Africa.^45,46^ The causes of death, including deaths following withdrawal of life-sustaining therapy, were not evaluated in this trial.

There was a significant reduction in therapy intensity in Salovum^®^-treated patients on Day 3 for participants that survived for more than 30 days, with a median of 4 in the Salovum^®^ group versus 8 in the control group. Although not significant after correction for multiple comparisons, this may indicate that Salovum^®^ does not only prevent deaths but may also diminish the need for neurointensive care interventions.

There was no significant change in ICP either as mean or as time over 20, 22, 25 and 30 mmHg between the groups. In the present trial, several explanations can be proposed: (i) that the hourly measurements of ICP do not reflect the true variation of ICP; (ii) that the sample size is too small to detect significant changes in ICP; (iii) that the standard of care effectively controlled ICP in both groups; and (iv) that the mode of action after exogenous administration of AF in TBI patients does not only involve control of ICP. The high number of primary decompressive craniectomies in both Salovum^®^ and control groups could also have reduced ICP levels.

The cause of death in severe TBI has commonly been associated with uncontrollable ICP, specifically during the first 48 h after the initial trauma.^16,47^ Randomized trials have reported either that ICP monitoring reduces mortality and morbidity, or that it does not influence outcome.^48,49^ Since ICP management did not differ significantly between the groups, the effect of Salovum^®^ is likely due to other mechanisms.

The trial protocol did not specify a minimal time of treatment, but because of the requirement for ICP monitoring during trial intervention, the treatment length was dictated by the clinical indications for ICP monitoring. In both arms, the intervention time was slightly less than 4 days. This implies that an even shorter intervention time than that, over 5 days, applied in the previous case series of Salovum^®^ administration to patients with severe TBI, significantly reduces mortality.

Other factors that could be dependent on trial intervention, such as days in the NICU and days in hospital, were likewise similar between the treatment arms. This indicates that these parameters are neither correlated to, nor causative of, the reduced mortality. A high proportion of the population served by Tygerberg University Hospital is from a low socioeconomic background. Resource limitations dictate that only participants unable to be discharged are referred to a rehabilitation facility, which restricts short- and long-term follow-up. There was no difference in number of days spent in hospital between the groups. Relative risks for mortality were also assessed according to trauma mechanism, suggesting a lower risk of death from subdural haematomas compared with contusions; however, these differences were not statistically significant. We cannot exclude the possibility that such trends may reach statistical significance in a larger cohort.

As is common in many LMICs, resource limitations mean that, in almost all cases, only patients with a post-resuscitation GCS of 6–8 would qualify for admission to the NICU. To minimize the risk of an uneven distribution, we chose to include only these participants. We acknowledge that while the present trial may be generalizable to the population experiencing severe TBI in many LMICs, it may not necessarily reflect the situation in countries with more advanced neurointensive care resources. Such settings may admit patients with lower GCS scores and may use a more diverse care, thus introducing a greater degree of heterogeneity to the patient population.^50^

The study’s main advantage lies in its use of the 30-day mortality rate as an objective indicator of treatment efficacy, which is a widely accepted and utilized approach. The trial’s limitations include the relatively small sample size of 100 participants, which may restrict the generalizability of the findings in relation to TBI interventions. Additionally, the study did not collect data on outcomes beyond the predetermined 30-day period, and thus neurological recovery could not be assessed beyond this time point.

## Conclusion

Administration of Salovum^®^ in conjunction with conventional treatment to adults with severe TBI, in a randomized study conducted in a resource-limited LMIC area, resulted in a statistically significant decrease in mortality rates compared to the control group. While the exact mechanisms of action of Salovum and antisecretory factor remain unclear, the findings of this study indicate a substantial impact on TBI outcome. Further studies aiming to investigate the mechanisms by which AF acts are warranted. The favourable safety profile, ease of administration and promising efficacy of AF support further evaluation in multicentre randomized controlled trials.

## Supplementary Material

awag241_Supplementary_Data

## Data Availability

The datasets generated and/or analysed during the current study are available from the corresponding author on reasonable request.
