# Supplementary material for: Salovum® reduces mortality in isolated severe traumatic brain injury: a randomized phase II trial
Source: Brain. 2026 Sep 7;149(11):3780–8. doi: 10.1093/brain/awag241 (PMC13730581; doi:10.1093/brain/awag241)
Supplement: Supplementary file 1 [file awag241_supplementary_data.pdf]

**Supplementary Table I**

| **TBI IMPACT risk estimates** | | | | |
|---|---|---|---|---|
| | **Active**<br>**N=49** | **Placebo**<br>**N=51** | **Missing values (A/P)** | **p-value[1]** |
| Six mo. mortality risk | 13.5 | 13.4 | 8/11 | 0.73 |
| Imputation 1 | 13.3 | 13.1 | | 0,90 |
| Imputation 2 | 12.9 | 13.1 | 0/0 | 0.85 |
| Imputation 3 | 12.6 | 13.2 | 0/0 | 0.81 |
| Imputation 4 | 13.5 | 13.3 | 0/0 | 0.85 |
| Imputation 5 | 13.8 | 13.2 | 0/0 | 0.82 |
| Six mo. unfavorable outcome risk | 26.5 | 26.5 | 8/11 | 0.73 |
| Imputation 1 | 25.4 | 26.1 | 0/0 | 0.90 |
| Imputation 2 | 25.7 | 25.2 | 0/0 | 0.85 |
| Imputation 3 | 25.8 | 24.6 | 0/0 | 0.81 |
| Imputation 4 | 26.0 | 26.2 | 0/0 | 0.85 |
| Imputation 5 | 25.8 | 26.4 | 0/0 | 0.82 |

[1] Wilcoxon test
